# Reducing the arbitrary: fuzzy detection of microbial ecotones and ecosystems – focus on the pelagic environment

**DOI:** 10.1186/s40793-020-00363-w

**Published:** 2020-08-13

**Authors:** Antoine Bagnaro, Federico Baltar, Gretchen Brownstein, William G. Lee, Sergio E. Morales, Daniel W. Pritchard, Christopher D. Hepburn

**Affiliations:** 1 Department of Marine Sciences, University of Otago, Dunedin, New Zealand; 2 NIWA, University of Otago, Dunedin, New Zealand; 3 Department of Functional & Evolutionary Ecology, Center of Functional Ecology, University of Vienna, Vienna, Austria; 4 Manaaki Whenua, Landcare Research, Dunedin, New Zealand; 5 Department of Microbiology & Immunology, University of Otago, Dunedin, New Zealand; 6 Te Ao Tūroa, Te Rūnanga o Ngāi Tahu, Dunedin, New Zealand

## Abstract

**Background:**

One of the central objectives of microbial ecology is to study the distribution of microbial communities and their association with their environments. Biogeographical studies have partitioned the oceans into provinces and regions, but the identification of their boundaries remains challenging, hindering our ability to study transition zones (i.e. ecotones) and microbial ecosystem heterogeneity. Fuzzy clustering is a promising method to do so, as it creates overlapping sets of clusters. The outputs of these analyses thus appear both structured (into clusters) and gradual (due to the overlaps), which aligns with the inherent continuity of the pelagic environment, and solves the issue of defining ecosystem boundaries.

**Results:**

We show the suitability of applying fuzzy clustering to address the patchiness of microbial ecosystems, integrating environmental (Sea Surface Temperature, Salinity) and bacterioplankton data (Operational Taxonomic Units (OTUs) based on 16S rRNA gene) collected during six cruises over 1.5 years from the subtropical frontal zone off New Zealand. The technique was able to precisely identify ecological heterogeneity, distinguishing both the patches and the transitions between them. In particular we show that the subtropical front is a distinct, albeit transient, microbial ecosystem. Each water mass harboured a specific microbial community, and the characteristics of their ecotones matched the characteristics of the environmental transitions, highlighting that environmental mixing lead to community mixing. Further explorations into the OTU community compositions revealed that, although only a small proportion of the OTUs explained community variance, their associations with given water mass were consistent through time.

**Conclusion:**

We demonstrate recurrent associations between microbial communities and dynamic oceanic features. Fuzzy clusters can be applied to any ecosystem (terrestrial, human, marine, etc) to solve uncertainties regarding the position of microbial ecological boundaries and to refine the relation between the distribution of microorganisms and their environment.

## Background

Nature is not uniform but is comprised of patches [1]. Identifying the boundaries between ecosystems and their characteristics remains a fundamental challenge for biogeographers and ecologists ([2, 3] for a review). Information on the spatial distribution of communities and ecosystems is key to understand community dynamics [4], and local and global diversity patterns [5] as well as guiding conservation strategies and ecosystem risk assessment protocols [6]. Initial qualitative assessment of boundaries within or between ecosystems can be done to direct sampling effort [7], but this approach is restricted to environments where delineations are clear (e.g. forest patches, [8], seagrass meadows, [9], agricultural land, [10]). Many ecosystems challenge such *à-priori* designs by presenting no obvious patchiness [11, 12], gradualness [13, 14], by being dynamic [15, 16], because the kind of organisms under consideration are too small for visual assessment [17, 18], or a combination of all or some of the above. In these cases, the selection of threshold values, or the definition of arbitrary boundaries, is often required to classify the different components of the ecosystems. The definition of thresholds may be arguable [19, 20] and these values can vary as much as the system they define [21, 22]. Together, these uncertainties reduce the potential comparability between studies [23] and opportunities for broader investigations across ecosystems.

The marine pelagic environment and its microbial communities offer an important example of this problem. On one hand, advances in satellite imagery [24] and recent sampling cruises [25, 26] reveal a patchy and dynamic ocean, with associated difficulties in the detection and delineation of water masses and ecosystems [11, 12]. On the other hand, the constant growth of sequencing techniques produces increasingly complex microbial data sets that challenge ecological and statistical interpretation [27].

Despite the challenges, microbial communities demand attention since they fulfil key functions in the oceans, including as nutrient and carbon recyclers [28]. Approximately one-half of the carbon fixed by marine autotrophs is directly processed by bacteria [28, 29]. Bacteria also facilitate the regeneration of nitrogen and phosphorus [30] and the release of iron [31], processes crucial to primary productivity [32]. Identifying spatial patterns in marine bacterial assemblages have the potential to reveal heterogeneity in oceanic productivity or biogeochemical patterns [33]. Recent studies did highlight associations between bacterioplankton communities and oceanographic features in the pelagic oceans [17, 18, 34–38] and in coastal waters along estuarine to marine transitions [39, 40]. Consistently identifying these associations requires methods to place the boundaries of ecosystems and to characterise the transitions between them (see [41, 42]). Transition zones between ecosystems, or ecotones, can play important ecological roles. They have been advocated as harbouring higher levels of productivity or diversity ([43] for a list of ecotone properties and further definition of ecotones) and to regulate the links between neighbouring systems [44, 45].

**Fig. 1:**
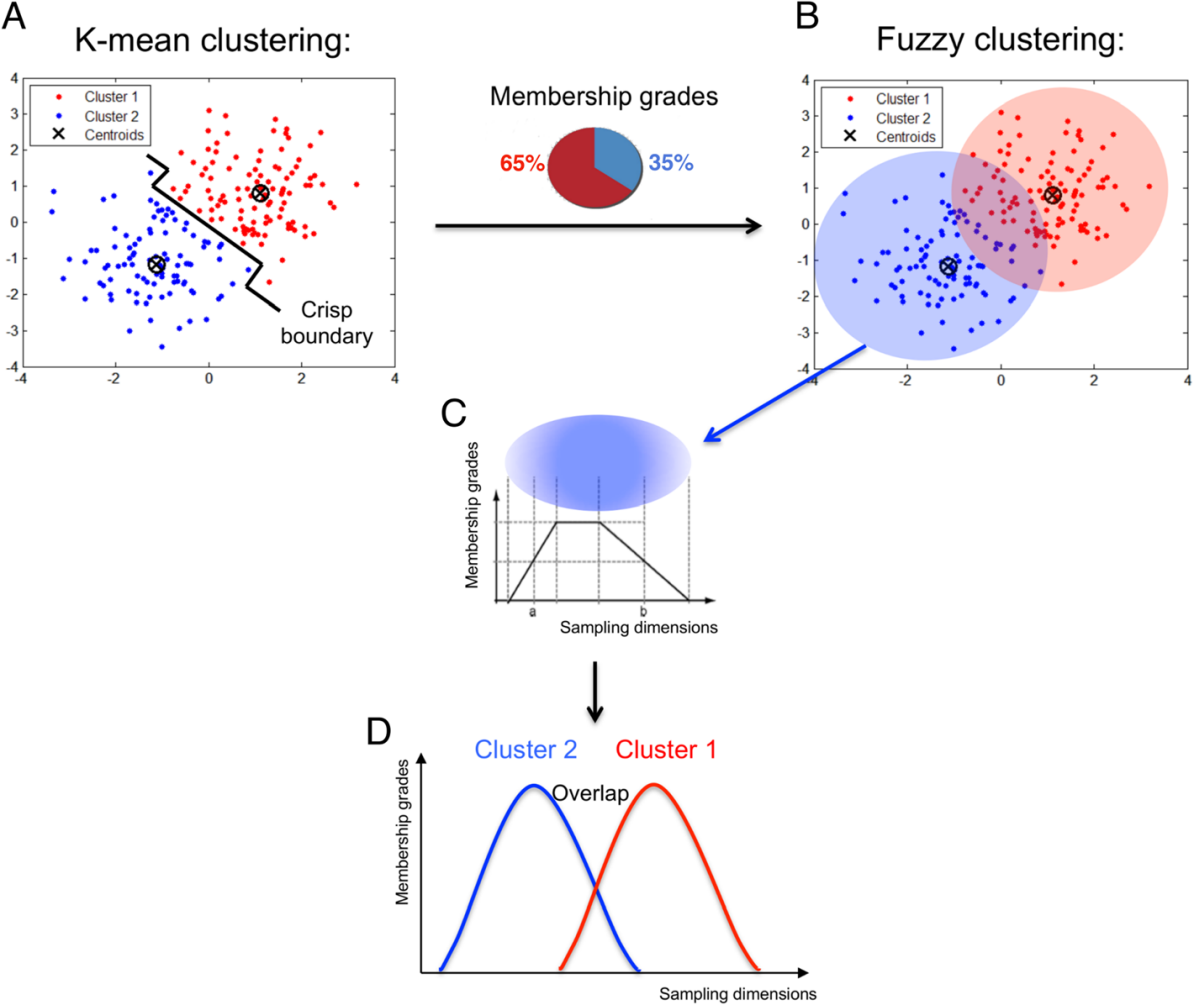
**a** Typical outputs of K-means clustering (2 clusters). The separation between the two clusters is crisp, as observations can only belong to one cluster, regardless of their actual distance to the cluster centroids. **b** Typical outputs of fuzzy clustering (same 2 clusters). Each observation now belongs to both clusters, according to their degree of similarity with each cluster centroid. This translates in their membership grades (e.g. 65% in cluster 1 and 35% in cluster 2). There is no strict boundary between the two clusters, as they now overlap. **c** Schematic representation of the membership grade profile of a single cluster over the spatial dimensions of the sampling. **d** Schematic representation of the overlap between the two clusters membership grade profiles over the spatial dimensions of the sampling

To examine ecosystems and their transitions in the pelagic environment, we used fuzzy clustering [46, 47], a technique that creates overlapping sets of clusters. A graphical explanation of the type of output generated by fuzzy clustering algorithms, in comparison to the more common k-means clustering, is given in Fig. 1. In fuzzy set theory, observations (usually sample sites in ecology) are assigned membership values in each fuzzy cluster in the range (0, 1), which expresses the degree to which the observation meets the definition of each cluster centroid [48]. In other words, the fuzzy cluster centroids correspond to the archetypical composition of the different communities [2], and each site is given membership values for each of the cluster, depending on how well the site composition reflects the centroid composition. Once plotted against the spatial dimensions of the gradient, the membership grades for a given cluster usually present a plateau surrounded by two declining edges, corresponding to a community core surrounded by two ecotones. As a consequence, the outputs of the analyses appear both structured (into clusters) and gradual (due to the overlaps), which aligns with the inherent continuity of natural systems, and particularly of the pelagic environment. Although not new, fuzzy logic approaches have been bypassed in ecological studies, where other types of gradient analyses prevail [10, 49]. However, rare applications have produced useful results [2, 50, 51]. The efficiency of fuzzy sets to describe ecological data has already been proven, when compared to ordinations such as canonical correspondence analysis (CCA) and distance-based redundancy analysis (DB-RDA) [48].

Here, fuzzy clusters were applied for the first time in the pelagic environment. We used the Munida Microbial Observatory Time Series (MOTS), a pelagic transect (for details see: Munida transect, [52, 53] for chemistry [17, 38, 54];), to explore the spatial associations of microbial communities (from OTUs based on 16S rRNA gene) and the surrounding oceanic structures (Sea Surface Temperature and Salinity) with a focus on ecotones.

## Materials and methods

### Study Site & Sampling

**Fig. 2:**
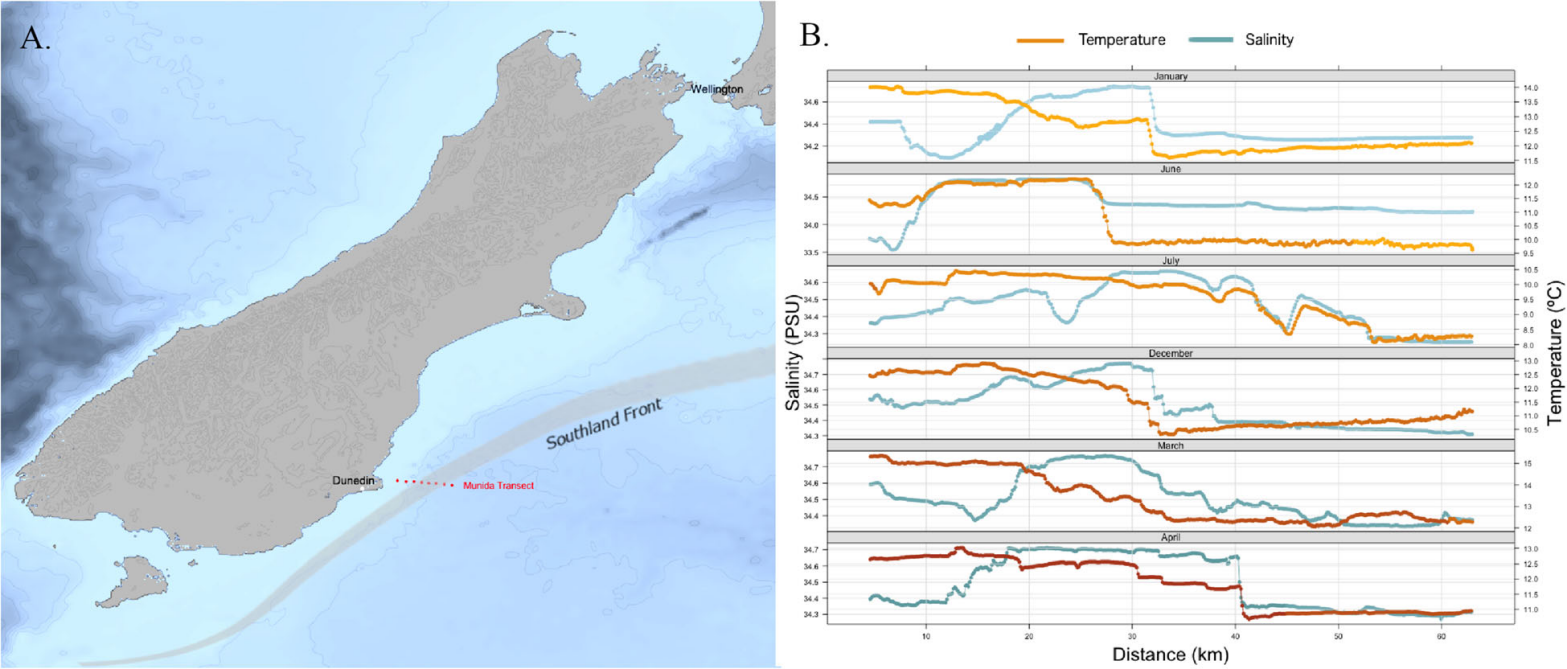
**a** Map of the sampling area. The red dots present the positions of the eight stations of the Munida transect. The grey shaded line marks the approximate location of the Southland front. **b** Sea Surface Temperature (orange lines) and Salinity (blue lines) were recorded directly inboard during each sampling cruise (right panels) along the whole transect (in kilometres from the shore) for each of the sampling month (January 2014, June 2014, July 2014, December 2014, March 2015 and April 2015)

The sampling was done along the Munida Microbial Observatory Time Series (MOTS, Fig. 2a). MOTS is a coastal transect crossing the subtropical front off the East Coast of the South Island, New Zealand. This 65 km long transect crosses multiple major oceanic features: neritic waters (NW), subtropical waters (STW), the subtropical front (FRONT), and sub-Antarctic waters (SAW). For this reason, it has been continuously investigated for almost two decades. The position and the width of these water masses are dynamic throughout the year and the subtropical front in particular has been extensively surveyed [22, 55]. It displays seasonal variations, being farther offshore during winter and closer inshore during summer (around 43 km and 27 km respectively, estimated from [22]). It is also narrowest during winter (15.07 km) and broadest during spring (23.88 km), with an average width of 8.36 km [56] or 18 km [22].

Sampling took place between January 2014 and April 2015 on the RV Polaris (see Fig. 2b for the 6 sampling months: January 2014, June 2014, July 2014, December 2014, March 2015 and April 2015). Continuous sea surface temperature (SST) and sea surface salinity (SSS) data were measured using a Sea Bird SBE45 thermosalinograph and associated SBE38 remote temperature sensor, with position information appended from the vessel GPS system.

Microbial communities were determined at each of the 8 stations of the MOTS from surface samples (2 m below the surface) and collected in 5 l acid-rinsed plastic bottles and then filtered (0.5–0.8 l) through 0.22 μm polycarbonate filters (Millipore). Duplicate samples for DNA extractions were taken together with samples for chlorophyll-a (Chl-a) analysis from each station [17, 38].

DNA was extracted separately from each filter using a PowerSoil® DNA Isolation Kit (MoBio, Carlsbad, CA, USA) and the manufacturer’s protocol. Bead beating was performed using a Geno/Grinder for 2 × 15 s and the final elution was done using 50 μL of solution C6 (sterile elution buffer, 10 mM Tris). DNA concentration was measured using a Nanodrop Spectrophotometer from Thermo Fisher. Amplicon libraries were generated using the Earth Microbiome Project barcoded primer set (V4 Primer 806R) and conditions [57]. All samples (independent replicates) were run on an Illumina Miseq run.

All amplicon data was processed using QIIME 1.9.1 and quality filtered using default parameters [57] with all fragments kept being 151 bp. Samples with less than 10,000 sequences were removed. Sequences were grouped into operational taxonomic units (OTUs) at 97% similarity. Open-reference OTU picking was carried out using the SILVA 119 release reference library and UCLUST [58]. Taxonomic assignments were performed using BLAST and the SILVA reference database. Multiple rarefactions (10) were performed to a depth of 10,000 sequences per sample and the files were merged to create an averaged OTU table. All data in the merged biom file were rounded prior to downstream analysis using the phyloseq package [59] in R (R Core Team 2019, [60]). Relative abundance (%) was calculated as the number of reads matching a particular OTU relative to the total number of reads. The data were then averaged for each of the sampling station (8 stations per month over 6 months) according to their positions along the transect (expressed in kilometres from the coastline) in order to build the microbial community matrices used for the statistical analysis.

### Statistical analysis

Fuzzy clusters (Fuzzy C-Means (FCM)) were constructed independently based on either the continuous temperature and salinity data (for water mass identification) or the microbial OTU data (for microbial communities identification) (fanny function, cluster package, [61], complemented by the vegclust function of the vegclust package, [47], to extract the fuzzy clusters centroid composition). The final number of clusters was chosen through an iterative process, with regard to the normalized partition coefficient and the normalized partition entropy [47]. Due to mathematical limitations, the number of fuzzy clusters cannot exceed n/2 − 1, where n is the total number of observations. This limitation meant that it was not possible to compute more than 3 fuzzy clusters out of the 8 sampled stations for the microbial communities. We therefore could not test for the existence of a fourth (or more) cluster in the OTU data.

First and second derivatives were calculated for each cluster from the regression of membership score on distance along the transect. The local maximum of the first derivative was used to pinpoint the location of the transitions between main environmental or community types and to assess the abruptness of these transitions (maximum slopes). The interval between two local maxima or minima was used as a proxy for the width of the environmental or community types. The widths of the transitions were assessed as being the interval between two local maxima of the second derivatives. We extracted these values for each of the sampling months and each of the clusters and compared the values grouped per month, seasons and water masses or communities. The statistical significance of the differences between groups was assessed by non-parametric Kruskal-Wallis tests followed by Dunn’s test for multiple pairwise comparisons and Bonferroni *p*-values adjustments (dunn.test package, [62]).

We extracted the fuzzy cluster centroids compositions (with the vegclust package) for each of the community clusters. These centroids correspond to the prototypical vector of the clusters in the multidimensional space of species abundances and can be used to represent community types ([47] and references therein). Every “species” in the dataset is present in the cluster centroids, and the most representative ones can be kept to describe the taxonomic composition of the clusters [2]. We compared the evolution of the fuzzy clusters at increasing taxonomic resolutions (from phyla to OTUs, Fig. SI.2). Further analyses on community composition were then conducted at the OTU level, to achieve a finer level of description [63]. Due to the high number of sampled OTUs, these results are presented as networks (qgraph package, [64]), in which the links correspond to the number of shared OTUs (OTU identities) between clusters. In order to avoid arbitrary decisions, a range of different thresholds was used to select the most representative OTUs in each cluster (ranging from 25 to 1% most representative, see supplementary information, Fig. SI.3A-3F). Networks often have several and equally correct graphical representations (in terms of where the nodes and edges should be drawn). We used the spinglass algorithm (repeated over 1000 networks, igraph package, [65]) to determine the actual number of communities in the network.

## Results

### Water masses and Bacterioplankton communities

**Fig. 3:**
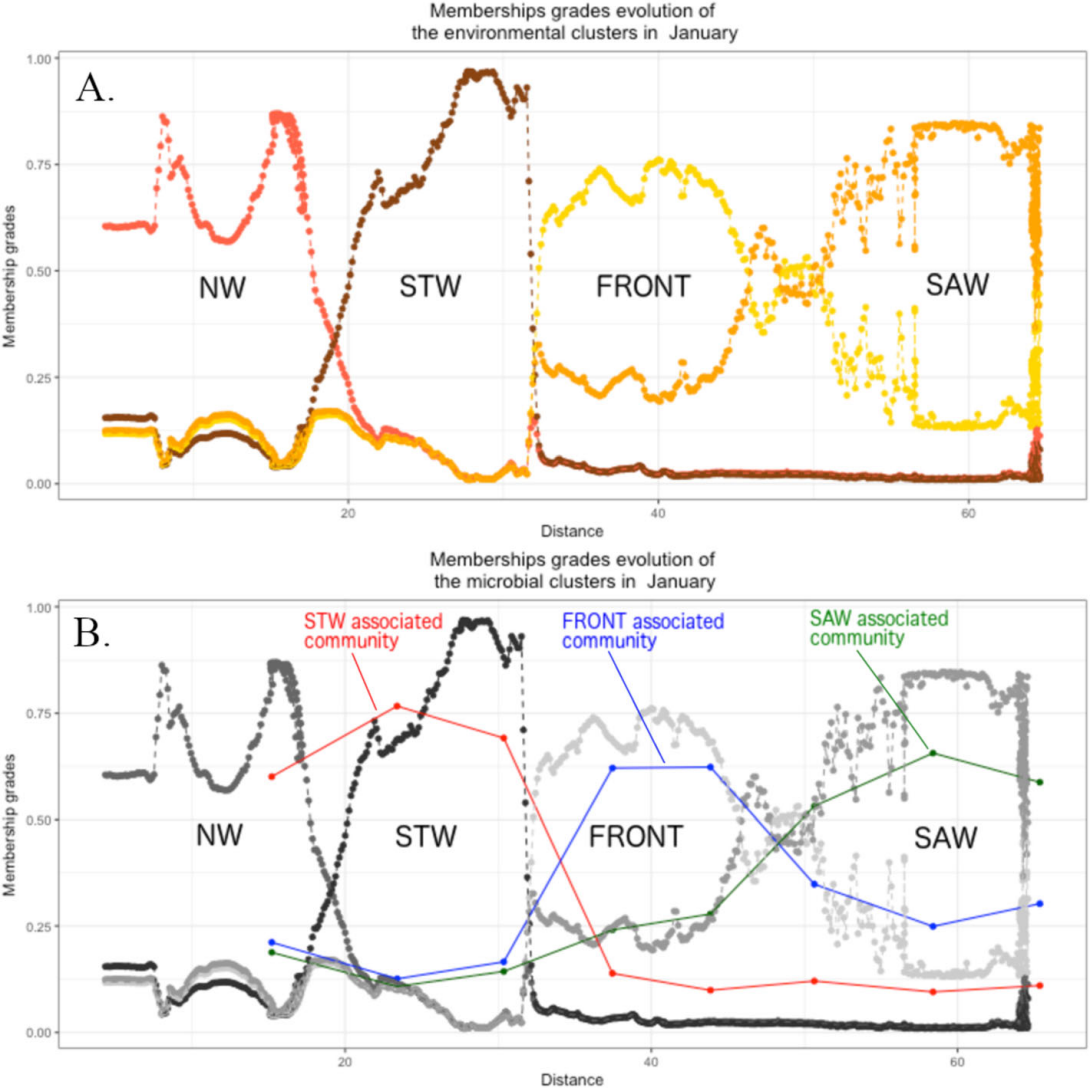
Example of the evolution of the fuzzy membership grades along the Munida transect (in kilometres from the shore) in January 2014. **a** The clusters based on environmental parameters (Surface temperature and Salinity) describe the different water masses: Neritic waters (red), Sub-Tropical waters (brown), Front (yellow) and Sub-Antarctic waters (orange). **b** Evolution of the microbial community clusters (based on OTUs) along the transect. The environmental clusters are kept in shades of grey for easiness of comparison. The evolution of the fuzzy membership grades for the other sampling months can be found in the SI, Fig. 1A to F

The continuous retrieval of temperature and salinity data allowed for a precise recording of the main water parameters along the Munida transect. The positions of the water masses could be directly approximated from the evolutions of SST and salinity (Fig. 2b) according to several thresholds ([55] for a complete list of values). The environmental fuzzy clusters, based on these two parameters, consistently described these water masses (Fig. 3a and Fig. SI. 1), and mean values for SST and SSS could be extracted from the fuzzy cluster centroids. Neritic waters (NW), characterised by high temperatures and low salinities (13.15 ± 1.25 °C, 34.32 ± 0.28 PSU), were the first to be crossed by the transect and finished between 9 and 19 km offshore depending on the sampling month. Subtropical waters (STW), characterised by higher temperatures and salinities (12.32 ± 0.93 °C, 34.67 ± 0.08 PSU), were next and continued until 27 to 32 km offshore. The subtropical front itself was described as a separate cluster by the FCM (11.30 ± 0.88 °C, 34.42 ± 0.13 PSU), with a width between 11 km and 22 km (for an average of 17 km), and occurred between the end of the STW and the beginning of the sub-Antarctic waters (SAW), which began between 41 and 53 km offshore. The low temperatures and salinity values, characteristic of the SAW (10.78 ± 1.32 °C, 34.31 ± 0.06 PSU), then continued until the end of the transect. The values given here are intervals, to account for the shape of the FCM and the monthly variability. We could not depict any clear seasonal patterns in the positions of the water masses (see supplementary information, Fig. SI. 1A-1F).

**Fig. 4:**
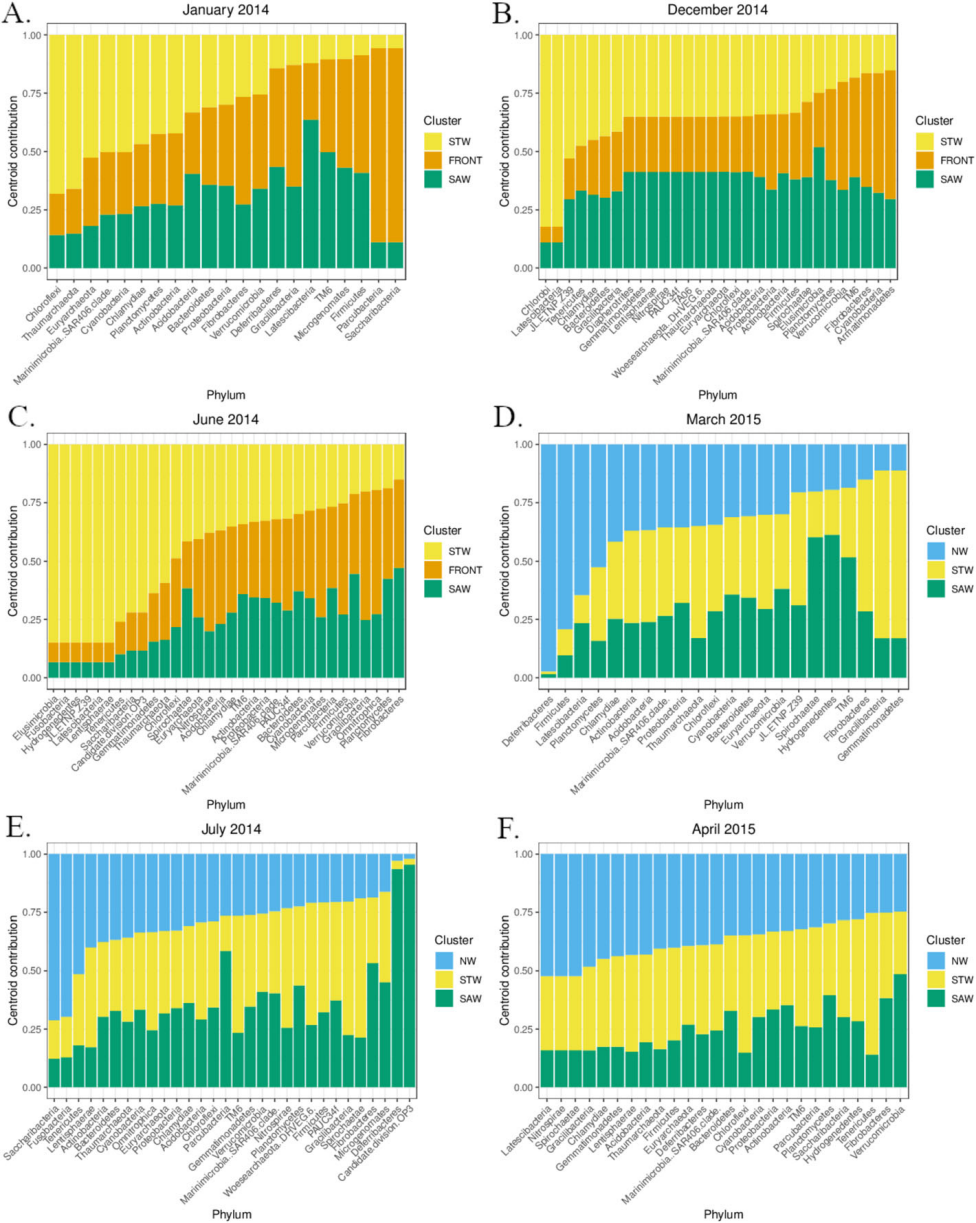
Barcharts with the centroid contributions of the sampled phyla in the different fuzzy clusters for each sampling month (**a** to **f**). The water mass affiliations of the microbial clusters are presented in blue (neritic waters), yellow (subtropical waters), orange (subtropical front) and green (subantarctic waters)

The FCM also described a spatial segregation of bacterioplankton communities along the transect (Fig. 3b and Fig. SI. 1). Due to the number of sampling stations and to computational constraints, the number of possible clusters was limited to three. Nevertheless, they were spatially organised and their positions coincided with the position of particular water masses. As such, it was possible to associate the bacterioplankton communities with the water masses in which they occurred. The STW and SAW could always be linked to a distinct microbial community, however the NW (present in July, March and April) and the subtropical front (present in January, June and December) inconsistently fluctuated between distinct and shared community clusters with bordering water masses. The relative positions of the fuzzy clusters did not vary dramatically depending on the taxonomic accuracy, but their spatial fit with the environmental clusters seemed to improve with increasing taxonomic resolution (Fig. SI. 2). The phylum composition of the fuzzy cluster centroids varied between months (Fig. 4). Some phyla (e.g. *Euryarchaeota*, *Verrucomicrobia*, *Planctomycetes* and *Bacteroidetes*) were consistently scoring intermediate values in each cluster, indicating ubiquitous distribution. Other phyla were more consistently associated with a single cluster at a time (e.g. *Latescibacteria* and *Tenericutes*, in NW or STW) and their spatial distribution therefore coincided with distinct water masses.

The overlaps and shapes created by the evolution of the FCM membership grades formed overlaps and areas of more or less mixed conditions between the different clusters. This representation offers a straightforward way to visualise the spatial organisation – or patchiness – of the environment from multivariate data. The status of boundary data, i.e. of data located in the outer part of the clusters, can therefore be more easily addressed.

### Transition patterns

**Fig. 5:**
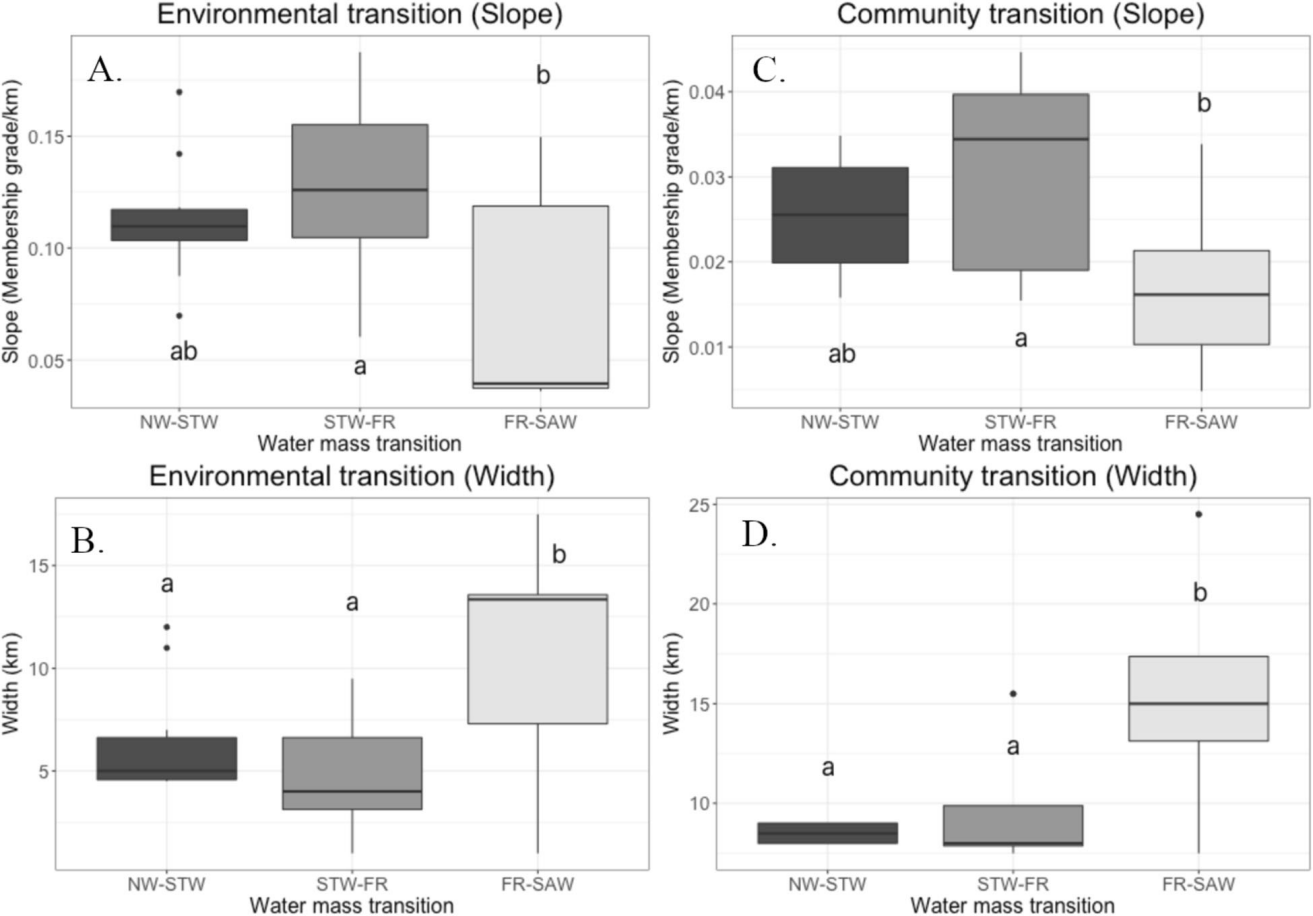
Abruptness (slope, panels **a** and **c**) and spatial extent of the transition (panels **b** and **d**) between the different water masses (**a** and **b**) and their associated communities (**c** and **d**). The small letters indicate the statistical significance in each panel (Dunn’s test with Bonferroni adjustments, *n* = 6, *p-value* < 0.05)

The characteristic (i.e., abruptness and the extent, here measured as slope and width) of the environmental transition zones along the transect were estimated (Fig. 5a-b). Overall, the transitions between the STW and the subtropical front were steeper and shorter than the transition from the subtropical front to the SAW (respectively × 1.69 steeper and × 2.29 km shorter on average, assessed with Kruskal-Wallis rank sum test, *p*-value < 0.05, *n* = 12), whereas the characteristics of the transitions between the NW and the STW did not significantly differ from the transitions between the STW and the subtropical front. All these values were subjected to important variations form 1 month to the other but did not reveal any strong seasonal trends.

The shapes of the transitions between the microbial clusters were congruent with those between the environmental clusters (Fig. 4c-d). The transitions between the communities in the STW and the subtropical front were × 1.78 steeper and × 1.59 shorter than the transitions between the communities in the subtropical front and the SAW. These differences were statistically significant (Kruskal-Wallis rank sum test, *p-value* < 0.05, *n* = 6), meaning that the environmental transitions and ecotones between the STW and the subtropical front were sharper and shorter than the ones between the front and the SAW. The transitions between the NW and the STW were not statistically different from the transitions from the STW and the subtropical front.

### System dynamic and relations between water masses and microbial communities

**Fig. 6:**
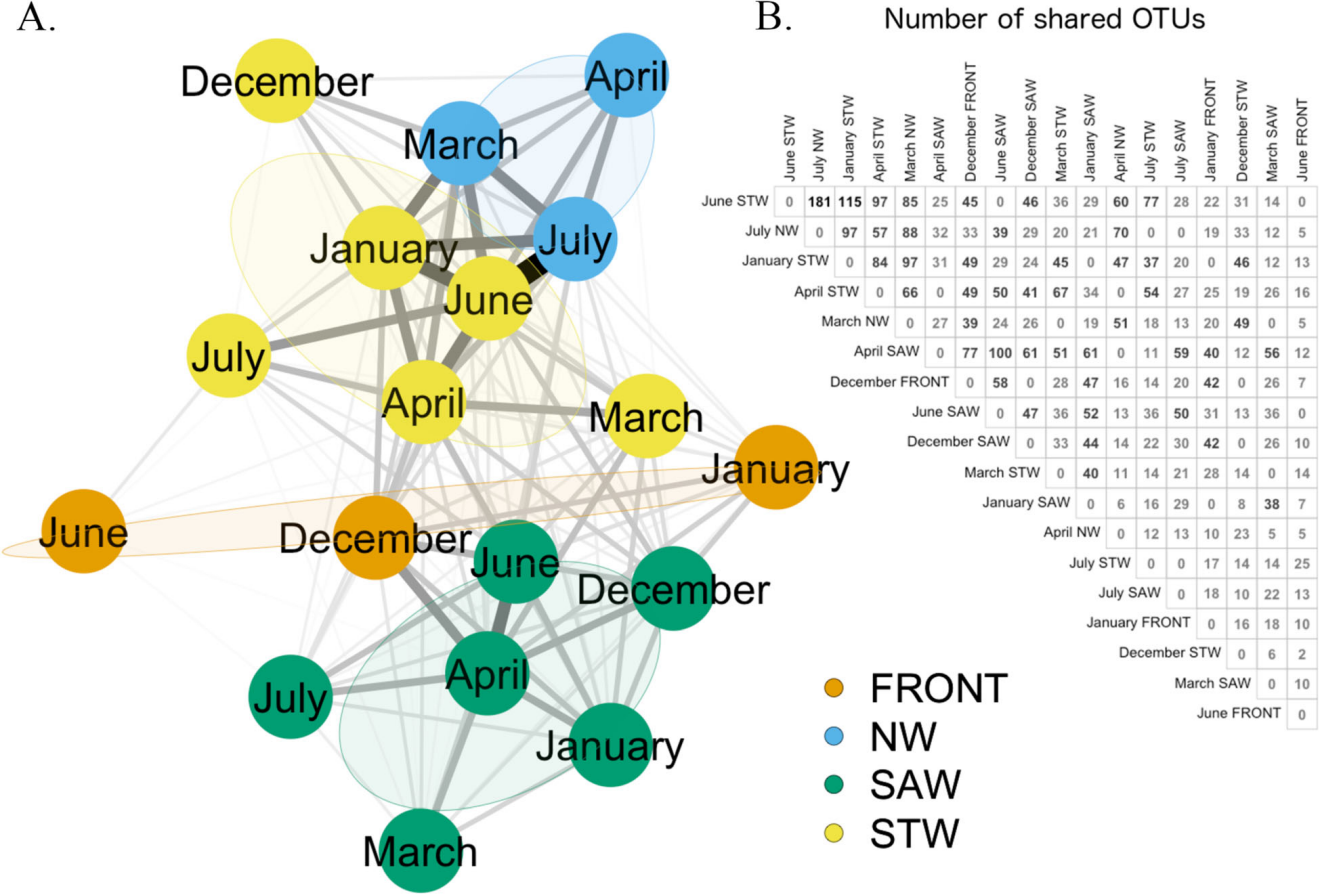
**a** Unweighted network representation of the relationship between the fuzzy community cluster centroids (defined for this network as the pool of OTUs that have a membership grade of 0.85 or more inside the cluster). The nodes have been coloured according to the water masses. The width of the links is proportional to the number of shared OTUs, which are reported on the correlation matrix (panel **b**)

As mentioned above, the variations in the positions and width of the microbial clusters matched those of the water mass (i.e. environmental) clusters. A network analysis (Fig. 6a) showed that these spatial associations also corresponded to a higher relatedness of the microbial communities that were representative of a particular water mass throughout the sampling period. As such, the links between the microbial communities that belonged to the same water masses were stronger than the links uniting water masses of different origins.

A visual analysis of the network structure reveals three different groups (i.e., the NW, STW and SAW associated communities), whereas the subtropical front communities are poorly linked with each other but draw a gap between the NW and STW communities on one side and the SAW communities on the other. Nevertheless, the spinglass algorithm only detected 2 statistical communities in the network (one comprised of the NW and STW communities and the other of the SAW and frontal communities), with the rare occurrence of the subtropical front community in June as a third isolate (> 1% of the networks). Although not in a different group, the STW community in December often appeared slightly apart from the other STW communities.

Importantly, the number of OTUs that were present in each cluster centroid only accounted for a small portion of the total number of OTUs. Such proportions are indicative of the degree of uniqueness in the microbial communities, as only a fraction of the OTUs played a role in linking the clusters (number of common OTUs between cluster centroids presented in Fig. 6b). Each month harboured an average of 41.1% (± 4.7%) of the total number of OTUs over the whole sampling period, and shared 51.5% (± 4.5%) of these remaining OTUs with other months. In the example network presented here (threshold value of 0.85, Fig. 6a), an average of 16.3% (± 5.3%) of the total number of OTUs/cluster centroid were above the threshold and considered as representative of a community. The remaining OTUs were therefore too ubiquitous to be kept in one particular cluster centroid. Among the representative OTUs of each cluster, an average of 6.4% (± 1.0%) were shared. The majority of the OTUs were therefore either too ubiquitous to represent a particular cluster, or too specific to be found in more than one cluster.

## Discussion

### Method considerations, suitability and efficiency

In this study, we used a fuzzy clustering approach for the first time to unravel the patchiness and the associated ecotones of microbial communities in the pelagic environment. We demonstrate that fuzzy clustering provides an efficient tool to unravel the heterogeneity of apparently continuous environments, particularly when the use of multivariate data is required, as it is the case for water masses (temperature and salinity) and microbial communities. There are three main advantages. First, the obtained graphical representation respects the continuity of the original data. It thus avoids arbitrary decisions regarding the assignation of spatial boundaries between clusters [66]. Second, FCM can be applied both on species (here OTU) and environmental data, with similar graphical outputs, which highlight spatial associations and makes comparison more straightforward. Finally, information from the cluster centroids can be extracted. These sets of vectors can be interpreted as biological description of the clusters [2, 47] or as representative values in the case of environmental variables. By keeping the ecosystem description structured in patches, it offers the possibility to bring together studies centred on ecosystem cores and research on ecotones, two approaches that have often been treated separately, despite their relation to understand landscapes as dynamic and networked entities [67, 68]. Recognising that patches can grade into each other aligns with more recent ecological theories, such as network theory [69] and complexity theory [67], that recognise that exchanges, links and continuity between entities are crucial for the description and functioning of ecosystems. Moreover, several quantitative metrics can be extracted from the shape of the fuzzy clusters, such as the extent and strength of the mixing area (here measured as widths and rate of change). Modelling studies suggest that the sharpness of the environmental transition is a major factor in controlling the characteristics of the ecotone, however, there is still a lack of empirical data to calibrate these models [4]. Using FCM along gradients may provide some of the metrics needed to progress in this field and prove useful for the parameterisation of statistical models that aim to account for fluxes and exchanges between compartments [70].

One drawback of this approach is the determination of the correct number of fuzzy clusters. Some mathematical indices have been developed to support this decision process, but they sometime fail to indicate a clear optimum [47]. The number of clusters is also constrained by the number of observations, which may be limiting at low sampling intensity.

### Water masses and microbial communities

The high variability we observed in the positions of the different water masses between the successive cruises describes the study area as a very dynamic system, with a wide range of possible states. We do not report seasonal trends in the position of the front, but the lack of time averaging in this study may have captured ephemeral variations, such as eddies, plumes and meanders, that can hide seasonal trends or associated cycles (as revealed in longer time series, [22, 56]). Therefore, although seasonality affects the average position of the oceanic features in the area, the range of possible locations remains high throughout the year (as reported in [22, 71, 72]). Here, this can be seen in the July data where the temperature and salinity records were disturbed.

FCMs offer an alternative approach in oceanography, and contrast to existing techniques for oceanic jet detection in the oceans (that are mostly based on satellite imagery algorithms, the use of gradient thresholding methods, probability density functions and contour methods, see [72] for a review). These methods make use of additional physical parameters such as Sea Surface Height (SSH) that are further away from the field of biological oceanography. Although FCMs could also be applied on SSH data, the use of SST and SSS facilitate the comparison of the subtropical front with surrounding oceanic features, as salinity and temperature are the most common descriptors to define water masses. These descriptors are also ecologically meaningful [73, 74] and the FCM do not exclude the possibility of including additional ecological parameters to better understand the drivers of species and community distribution.

Patterns of association between plankton and oceanic circulation have been demonstrated at larger scales, mainly through ocean colour imagery (Chlorophyll a, but also with other pigments, proteins and lipids [18, 75–77]). The phylum composition of the different fuzzy cluster centroids highlighted a ubiquitous distribution of some of the phyla along the 60 km of the transect, among which the *Euryarchaeota* (methanogens), *Verrucomicrobia*, *Planctomycetes*, *Bacteroidetes* (known particle degraders, [78]) and SAR406. Many of these phyla have been linked with anaerobic metabolisms in deeper parts of the oceans [79]. Their presence in surface samples may either indicate a strong mixing of the surface waters with deeper layers, the ability of some of the members of these phyla to live in more oxic conditions, or their association with suboxic microhabitats in particles (see [80, 81]). Other phyla were more consistently associated with a single cluster at a time, e.g. *Latescibacteria* (NW in March and April 2015, STW in June 2014), *Tenericutes* (NW July 2014, STW in June 2014 and April 2015). These particular associations mostly happened in the nearshore sections of the transect and could indicate that the subtropical front and the SAW are poorly differentiated at the phylum level, comparatively to the NW and STW. Information on the basic metabolism and ecological properties of these microorganisms are still scarce [82] and drawing ecological conclusions from these patterns would still require too many unsupported assumptions, particularly as microbial taxonomic diversity may be a poor predicator of microbial functional diversity [83]. The spatial segregation of the fuzzy clusters was only slightly more pronounced at increased taxonomic resolution (Fig. SI. 2), which may suggest that members of the same phylum might share general ecological strategies [84]. Ecological communities are shaped by more than just the sum of individual responses to surrounding environmental conditions. They are usually stabilised by facilitative or mutualistic relationships (sometime centred around a few key species [85]). Genomic studies have suggested that many bacterial phyla, and in particular those with small genome sizes could be obligate synthrophs, depending on larger bacterial hosts (e.g. in the Candidate Phyla Radiation and TM6 [82, 86, 87]). Such microbial associations may introduce reinforcement loops in the composition of microbial community, particularly at taxonomic resolutions that capture more specialised relationships. Investigating the co-occurrence of particular taxa in environmental datasets could help exploring these associations [82].

To account for these microbial associations, the community networks were thus constructed at the highest taxonomic resolution (OTU). The consistency of the links that united the bacterial communities of a particular water mass demonstrate the existence of biogeographic patterns in pelagic microbial communities (see also [88, 89] for bacterial communities), even at local scales over few kilometres. These patterns may be driven by the dispersal limitations induced by water masses circulation or by “ecosystem filtering” (including biotic and abiotic filters). We nevertheless recognise that these drivers unequally affected the microbial communities. Many OTUs were ubiquitous in the study area and therefore unrelated to a particular cluster centroid, conversely many others were too rare, i.e. occurring in only one cluster, to play a role in the networks. Overall, only a small proportion of the OTUs explained the community variance. An average of 41% of the total number of OTUs sequenced over the whole sampling period were present per month and only 16.3% of that number were representative of a given cluster (the other 83.7% were too ubiquitous to represent a cluster). 6.4% were shared between clusters. These numbers can provide estimates for alpha and beta diversity (respectively defined as the diversity in a single community and the total dissimilarity between two or more communities, [90, 91]). If considered month per month, the high percentage of ubiquitous OTU advocate for low beta diversity between the water masses and a high alpha diversity in each water mass. Other microbial studies on land have found patterns of low beta diversity and high alpha diversity in microbial communities ([92] on soil microbiota), which is consistent with the results of this study. However, we point out that the low percentage of OTUs shared between the successive months suggests much higher beta diversity over time. In the case of microorganisms, their ability to evolve quickly may result in emergent patterns of beta diversity [93] and may provide an explanation for these findings. Inasmuch as we can tell from the limited data of this study, we advocate that time alone could be an important driver of microbial diversity patterns (along with dispersal limitations and environmental filtering) and that its effect on microbial diversity should be further isolated and explored, notably as it might provide a link between the slow-evolving macro-organisms and the fast-evolving microorganism diversity patterns.

Considering the study area, higher numbers of shared OTUs were reported between the microbial communities in the NW (and STW in a lesser extent) compared to the offshore communities (subtropical front and SAW, Fig. SI. 3), thus revealing more conserved communities over time. The strong influence of the Clutha River in the region, with its consistent inputs of freshwater and sediments, is responsible for the existence of a well differentiated NW, and is already known to support higher levels of primary productivity [94]. These terrestrial inputs may have had an additional stabilizing effect on the particle degrading bacterioplankton communities in the surface layers sampled in this study.

This study was indeed limited to the surface waters. Depth influences the water mass characteristics ([95], for New Zealand), and can drive microbial community differentiation, particularly on both side of the euphotic zone [96]. Mixing events however regularly occur and can lead to a homogenisation of the water column down to depths of around 100 m [22]. The conclusions of our study thus mainly apply to these depths.

### Transition patterns, oceanic front and ecotone

It is generally accepted that water mass properties change rapidly across the front, yet remain approximately constant along the front [71]. As such, frontal areas have been regarded as transition zones between water masses and create potential ecotones [17, 38]. The FCMs were able to isolate the front as a distinct environmental cluster. The physical characteristics of the transitions between the different water masses and microbial communities along the continental shelf revealed that the passing from the frontal waters to the SAW were more gradual and over longer distances than the other transitions, which is probably the consequence of the sub-Antarctic origin of the subtropical front that makes its composition more similar to the SAW (90% SAW and 10% STW, according to [97]).

Despite this similarity with the SAW, the subtropical front in this study often maintained specific microbial clusters, comprising relatively low numbers of shared OTUs with the surrounding water masses (Fig. SI. 3). The origin of these few shared OTUs appeared to be equally partitioned between the STW and the SAW, but remained marginal in the cluster composition, highlighting the emergence of a separate community. These particular microbial communities were especially evident over the summer months, when stronger gradients in nutrient concentrations and temperature prevail and when phytoplankton blooms may occur [22, 52]. In these conditions, phytoplankton dynamics may drive the emergence of a distinctive microbial community [17, 38, 98, 99]. By contrast, a microbial community associated with the less salty neritic waters replaced it during the winter and autumn months, as the gradients associated with the front weaken [22]. Nevertheless, the appearance of a front-associated microbial cluster in June is at odds with this interpretation and we cannot reject a detrimental effect of the limited number of possible microbial fuzzy clusters.

The subtropical front did act as a clear separator between the STW and the SAW. Its position as an ecotone or as a separate and distinct ecosystem remains debatable. Temporal – and possibly seasonal – patterns may be the main drivers for whether or not the front harbours a separate community, or is an ecotone. Transient ecotones have been reported in the terrestrial literature [100]. This distinction is important, as the exchanges between the STW and the SAW are likely to be impacted by the presence of a distinctive microbial community between them, as well as the capacity of the frontal area to support higher levels of productivity [101] and attract organisms belonging to higher trophic levels [102].

Our study revealed the existence of another ecotone between the coastal NW and the STW, with the persistence of a coastal microbial biome. The occurrence of coastal front associated with freshwater inputs have already been reported [103] and linked with distinct plankton communities [104]. Additionally, we demonstrate its influence on microbial communities, which has implications for primary productivity in coastal waters [28, 105]. Microbial ecotones may represent gradients of differing biochemical activities, where the frontal microorganism benefit from the combined access to different sources of nutrients, and thus overcome potential growth limitations. Oceanic fronts are usually presented as areas of enhanced productivity [55, 106] and our ability to pinpoint and characterise the pelagic environment as continuous and comprised of both patches and the transitions between them might prove crucial to understand the linkage between the different trophic levels in the oceans, in turn improving our understanding of global biogeochemical cycles [33, 107].

## Conclusion

The use of fuzzy clustering offers a novel way of integrating environmental and biotic entities in continuous representations, thus bringing together both patches and ecotones in ecosystem studies. Here, this technique allowed us to reveal the water mass organisation along the subtropical convergence zone off South New Zealand. In particular, we report the subtropical front as a distinct oceanic structure. Each water mass harboured distinctive bacterioplankton communities with high alpha diversity but low beta diversity although there was an important effect of time on microbial community composition. The characteristics of the ecotones between these communities matched the characteristics of the transitions between the different water masses, indicating that the mixing of the waters also resulted in the mixing of the microbial communities. It is noteworthy, that a pelagic oceanic front was used as a case study, but fuzzy clustering may be applied to a wide range of ecological systems and proves a powerful asset to resolve uncertainties regarding the patchiness along environmental gradients.

## Supplementary information

**Additional file 1.** Fig. SI.1: Variations of the membership grades of the environmental fuzzy clusters (in shades of black) and of the microbial clusters (in red, blue and green) along the Munida transect (in kilometres from the shore) for the different month of the sampling. The microbial communities have been labelled according to the water mass that they occupy. The computations of the two sets of clusters (environmental and microbial) were independent thus their spatial correspondence is incidental. Fig. SI.2: Evolutions of the fuzzy clusters at the different sampling time (A to F) depending on the taxonomic resolution of the microbial data. Taxonomic levels spans from Phylum (lowest resolution, row 1) to OTUs (highest resolution, row 4), with order (row 2) and genus (row 3) as intermediate taxonomic resolutions. The water masses fuzzy clusters are presented in the background and are identical to Fig. SI.1. The relative positions of the transitions between the microbial clusters are hardly affected by the taxonomic resolution. Their shapes, however, are and the microbial clusters match the environmental cluster best at increased taxonomic resolutions. Fig. SI.3: Network representations of the number of shared OTUs between the microbial communities in association to their water masses (colours) and the sampling month (labels). The thicknesses of the links are proportional to the number of shared OTUs. The exact numbers are reported on the matrix on the right of each network. The coloured ellipse areas correspond to the 95% probability region for the water masses in the networks. Note that the higher the threshold, the weaker the links. For the highest thresholds (0.95 and 0.99), some nodes are not linked to any others and their positions should not be interpreted.

## Data Availability

The raw sequence reads have been deposited to the NCBI Short Read Archive (SRA) under BioProject accession number PRJNA430292 (https://www.ncbi.nlm.nih.gov/bioproject/PRJNA430292/). The datasets used during the current study are available as supplementary files, as well as the R codes used for the data analysis, according to the description given in the materials & Method section.
