# Supplementary material for: Reducing the arbitrary: fuzzy detection of microbial ecotones and ecosystems – focus on the pelagic environment
Source: Environ Microbiome. 2020 Aug 13;15:16. doi: 10.1186/s40793-020-00363-w (PMC8066478; doi:10.1186/s40793-020-00363-w)
Supplement: Supplementary file 1 — Additional file 1. Fig. SI.1: Variations of the membership grades of the environmental fuzzy clusters (in shades of black) and of the microbial clusters (in red, blue and green) along the Munida transect (in kilometres from the shore) for the different month of the sampling. The microbial communities have been labelled according to the water mass that they occupy. The computations of the two sets of clusters (environmental and microbial) were independent thus their spatial correspondence is incidental. Fig. SI.2: Evolutions of the fuzzy clusters at the different sampling time (A to F) depending on the taxonomic resolution of the microbial data. Taxonomic levels spans from Phylum (lowest resolution, row 1) to OTUs (highest resolution, row 4), with order (row 2) and genus (row 3) as intermediate taxonomic resolutions. The water masses fuzzy clusters are presented in the background and are identical to Fig. SI.1. The relative positions of the transitions between the microbial clusters are hardly affected by the taxonomic resolution. Their shapes, however, are and the microbial clusters match the environmental cluster best at increased taxonomic resolutions. Fig. SI.3: Network representations of the number of shared OTUs between the microbial communities in association to their water masses (colours) and the sampling month (labels). The thicknesses of the links are proportional to the number of shared OTUs. The exact numbers are reported on the matrix on the right of each network. The coloured ellipse areas correspond to the 95% probability region for the water masses in the networks. Note that the higher the threshold, the weaker the links. For the highest thresholds (0.95 and 0.99), some nodes are not linked to any others and their positions should not be interpreted. [file 40793_2020_363_MOESM1_ESM.pdf]

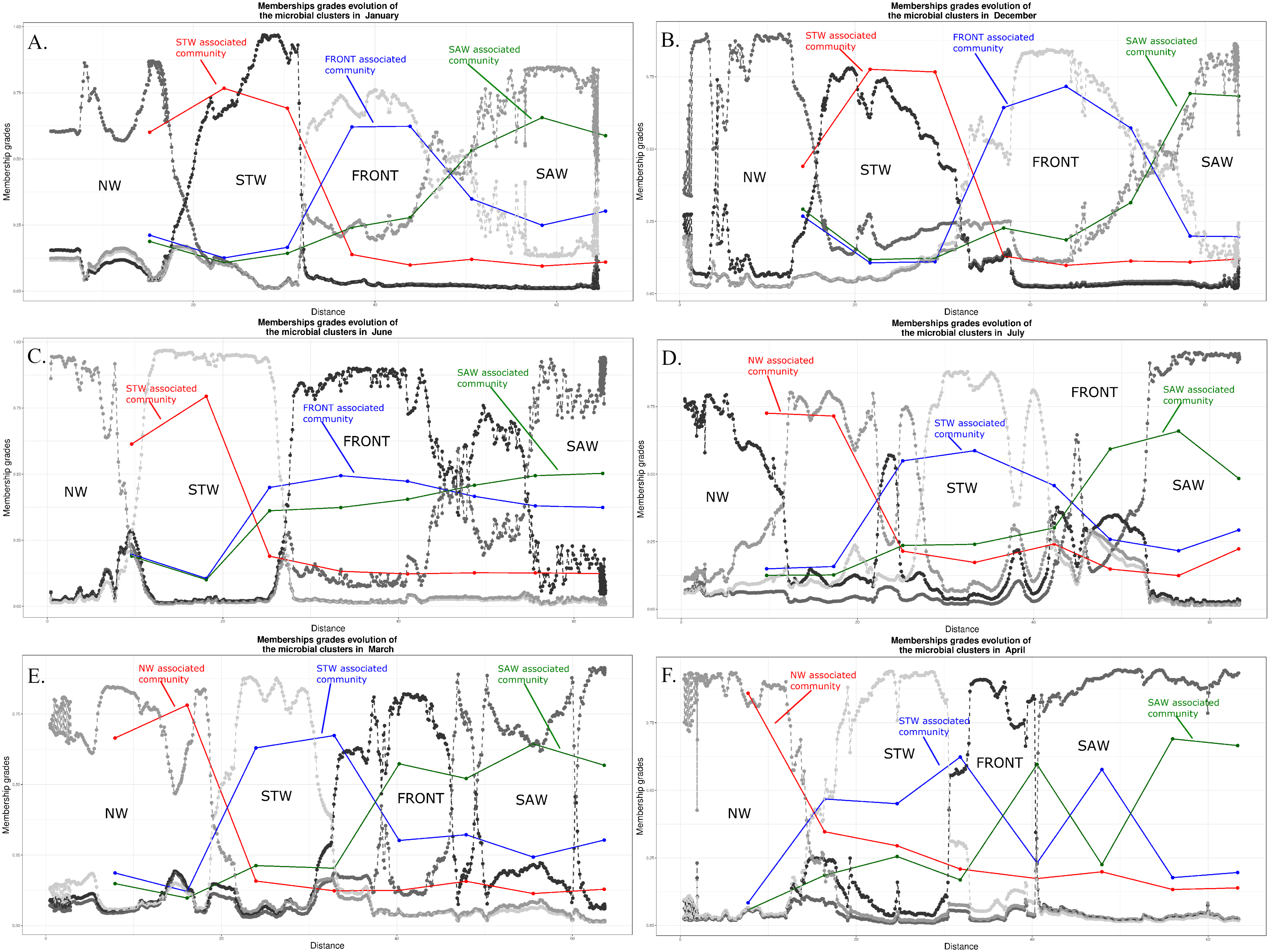
Memberships grades evolution of
the microbial clusters in January
A.
STW associated
community
FRONT associated
community
SAW associated
community
NW
STW
FRONT
SAW
Membership grades
Distance
Memberships grades evolution of
the microbial clusters in December
B.
STW associated
community
FRONT associated
community
SAW associated
community
NW
STW
FRONT
SAW
Membership grades
Distance
Memberships grades evolution of
the microbial clusters in June
C.
STW associated
community
FRONT associated
community
SAW associated
community
NW
STW
FRONT
SAW
Membership grades
Distance
Memberships grades evolution of
the microbial clusters in July
D.
NW associated
community
STW associated
community
SAW associated
community
NW
STW
FRONT
SAW
Membership grades
Distance
Memberships grades evolution of
the microbial clusters in March
E.
NW associated
community
STW associated
community
SAW associated
community
NW
STW
FRONT
SAW
Membership grades
Distance
Memberships grades evolution of
the microbial clusters in April
F.
NW associated
community
STW associated
community
SAW associated
community
NW
STW
FRONT
SAW
Membership grades
Distance

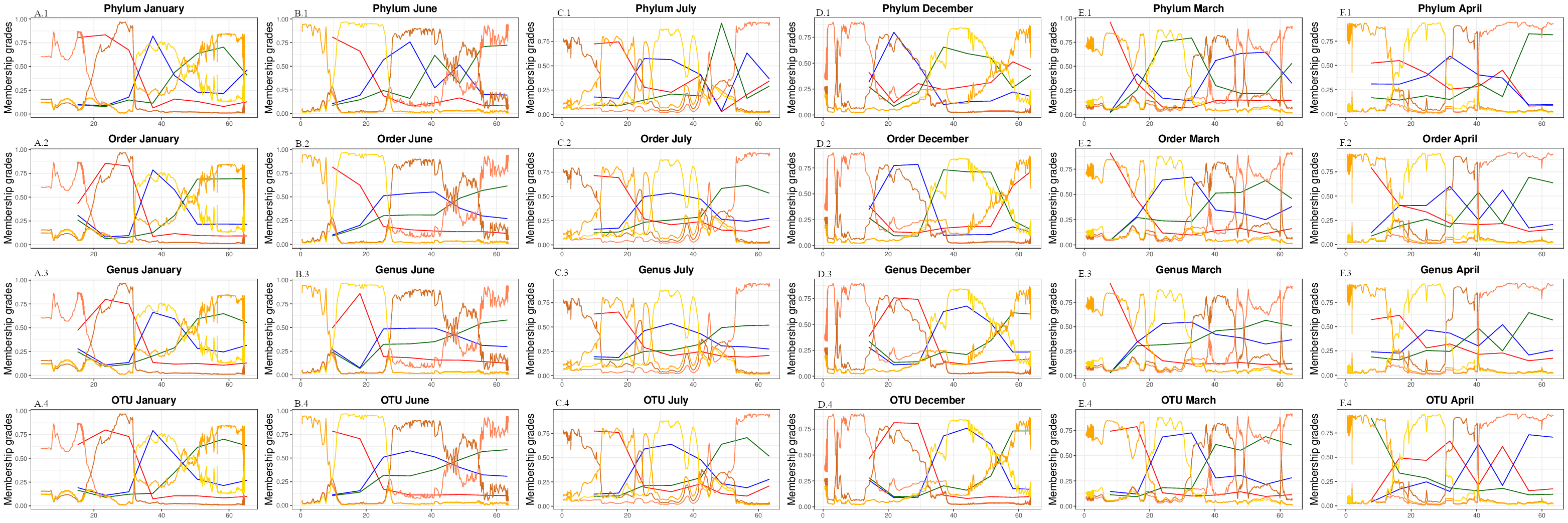

A.1 Phylum January
B.1 Phylum June
C.1 Phylum July
D.1 Phylum December
E.1 Phylum March
F.1 Phylum April
A.2 Order January
B.2 Order June
C.2 Order July
D.2 Order December
E.2 Order March
F.2 Order April
A.3 Genus January
B.3 Genus June
C.3 Genus July
D.3 Genus December
E.3 Genus March
F.3 Genus April
A.4 OTU January
B.4 OTU June
C.4 OTU July
D.4 OTU December
E.4 OTU March
F.4 OTU April
Membership grades

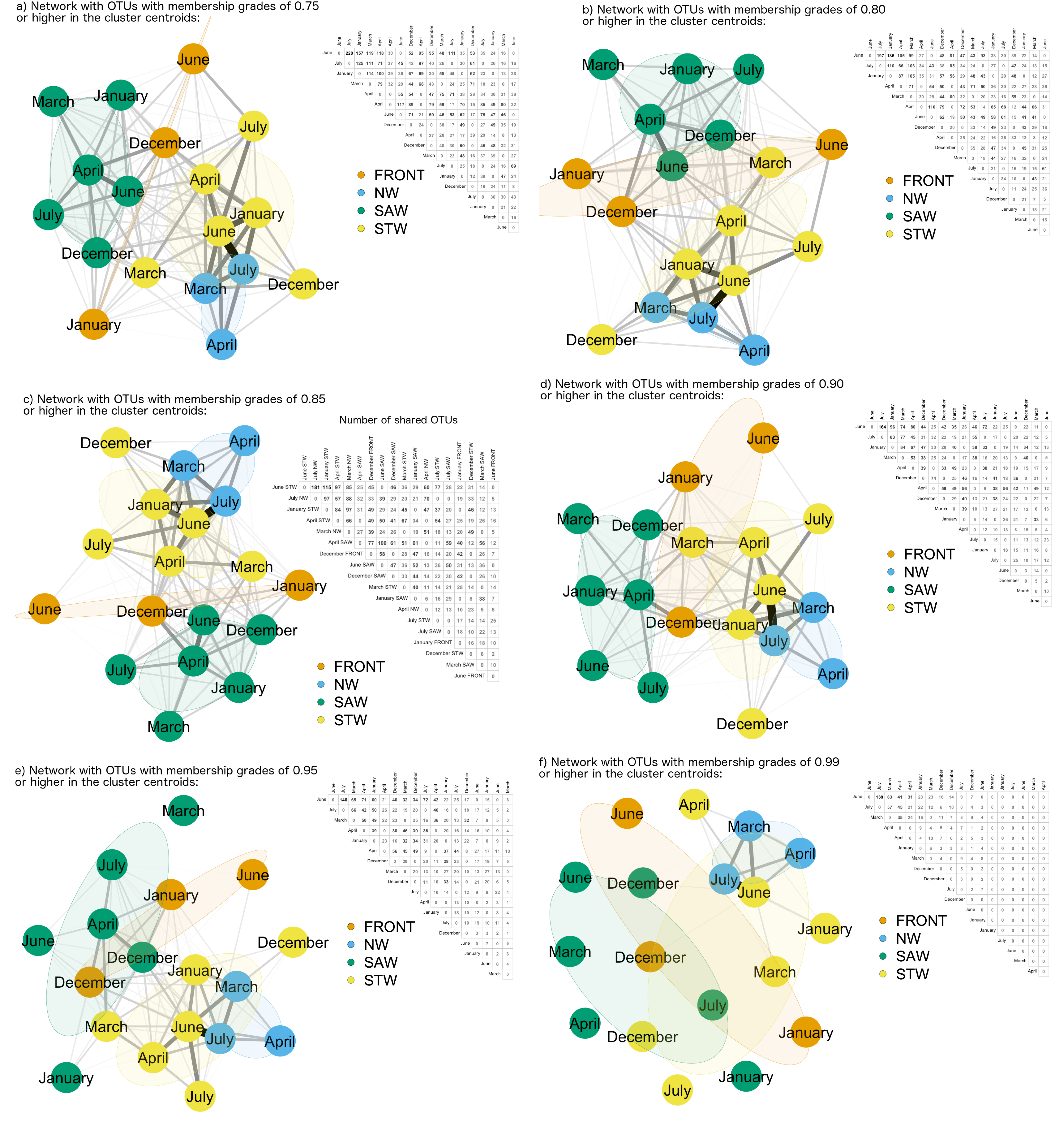
a) Network with OTUs with membership grades of 0.75 or higher in the cluster centroids:
b) Network with OTUs with membership grades of 0.80 or higher in the cluster centroids:
c) Network with OTUs with membership grades of 0.85 or higher in the cluster centroids:
Number of shared OTUs
d) Network with OTUs with membership grades of 0.90 or higher in the cluster centroids:
e) Network with OTUs with membership grades of 0.95 or higher in the cluster centroids:
f) Network with OTUs with membership grades of 0.99 or higher in the cluster centroids:
FRONT
NW
SAW
STW
